# Supplementary material for: Divergent patterns of cognitive decline in preclinical Alzheimer's disease: Implications for secondary prevention trials
Source: Alzheimers Dement. 2026 Apr 21;22(4):e71366. doi: 10.1002/alz.71366 (PMC13099594; doi:10.1002/alz.71366)
Supplement: Supplementary file 1 — Supporting Information [file ALZ-22-e71366-s002.pdf]

Divergent patterns of cognitive decline in preclinical Alzheimer's disease: implications for secondary prevention trials

Runpeng Li Oliver Langford Philip S. Insel Reisa A. Sperling
Rema Raman Paul S. Aisen Michael C. Donohue

Supplementary Material

Supplementary Material

	Stable (N=312)	Slow decliner (N=73)	Fast decliner (N=42)	Total (N=427)	p value
Group					0.004
- LEARN	52 (16.7%)	3 (4.1%)	0 (0.0%)	55 (12.9%)	
- Placebo	131 (42.0%)	34 (46.6%)	23 (54.8%)	188 (44.0%)	
- Solanezumab	129 (41.3%)	36 (49.3%)	19 (45.2%)	184 (43.1%)	
Age, mean (SD), y	71.15 (4.51)	73.74 (4.83)	72.80 (5.65)	71.76 (4.78)	< 0.001
Sex, No. (%)					0.009
- Male	126 (40.4%)	40 (54.8%)	11 (26.2%)	177 (41.5%)	
- Female	186 (59.6%)	33 (45.2%)	31 (73.8%)	250 (58.5%)	
Race, No. (%)					0.426
- N missing	3	0	1	4	
- Am. Indian or Alaska Native	1 (0.3%)	0 (0.0%)	0 (0.0%)	1 (0.2%)	
- Asian	12 (3.9%)	2 (2.7%)	4 (9.8%)	18 (4.3%)	
- Native Hawaiian or Other PI	0 (0.0%)	0 (0.0%)	0 (0.0%)	0 (0.0%)	
- Black or African Am.	7 (2.3%)	4 (5.5%)	0 (0.0%)	11 (2.6%)	
- White	287 (92.9%)	67 (91.8%)	37 (90.2%)	391 (92.4%)	
- Unknown or Not Reported	2 (0.6%)	0 (0.0%)	0 (0.0%)	2 (0.5%)	
Ethnicity, No. (%)					0.842
- Hispanic or Latino	5 (1.6%)	1 (1.4%)	1 (2.4%)	7 (1.6%)	
- Not Hispanic or Latino	302 (96.8%)	70 (95.9%)	41 (97.6%)	413 (96.7%)	
- Unknown or Not reported	5 (1.6%)	2 (2.7%)	0 (0.0%)	7 (1.6%)	
Education, mean (SD), y	16.24 (2.89)	16.16 (2.66)	16.05 (2.70)	16.21 (2.83)	0.904
APOE4, No. (%)					< 0.001
- non-carrier	166 (53.2%)	22 (30.1%)	11 (26.2%)	199 (46.6%)	
- carrier	146 (46.8%)	51 (69.9%)	31 (73.8%)	228 (53.4%)	
PACC, mean (SD)	0.51 (2.48)	-1.20 (2.57)	-2.57 (3.20)	-0.09 (2.77)	< 0.001
Amyloid PET, mean (SD), CL	47.04 (30.54)	77.33 (38.73)	86.23 (34.78)	56.07 (35.74)	< 0.001
P-tau217, mean (SD), U/ml	0.22 (0.10)	0.33 (0.17)	0.43 (0.20)	0.26 (0.15)	< 0.001
Hipp. atrophy, mean (SD), z-score	0.03 (0.83)	0.80 (0.85)	0.96 (0.79)	0.25 (0.91)	< 0.001
Tau PET, mean (SD), SUVr	1.08 (0.07)	1.13 (0.08)	1.23 (0.13)	1.10 (0.09)	< 0.001
CDR Prog., No. (%)					< 0.001
- CDR Non-progressor	217 (69.6%)	18 (24.7%)	2 (4.8%)	237 (55.5%)	
- CDR Progressor	95 (30.4%)	55 (75.3%)	40 (95.2%)	190 (44.5%)	

Supplementary Table S1: **Baseline demographic, clinical, and biomarker characteristics by latent class of cognitive decline with tau PET.** Baseline characteristics of participants classified by the latent class mixed model (LCMM) of Preclinical Alzheimer Cognitive Composite (PACC) trajectories. Classes represent distinct longitudinal cognitive patterns: stable, slow decliner, and fast decliner. Continuous variables are presented as mean (SD); categorical variables as No. (%). **Abbreviations:** APOE = apolipoprotein E; PET = positron emission tomography; P-tau217 = plasma phosphorylated tau 217; SUVr = standardized uptake value ratio; U/mL = arbitrary units proportional to assay signal; Am. = American; PI = Pacific Islander; CDR = Clinical Dementia Rating; Prog. = Progressor. **Footnotes:** Amyloid PET and tau PET SUVr values represent mean cortical uptake relative to cerebellar reference region. Hippocampal atrophy are residualized for intracranial volume and z-scored. Education reported in years of formal schooling. CDR Progressors were observed to have a CDR Global score greater than zero at two consecutive visits, or their last visit.

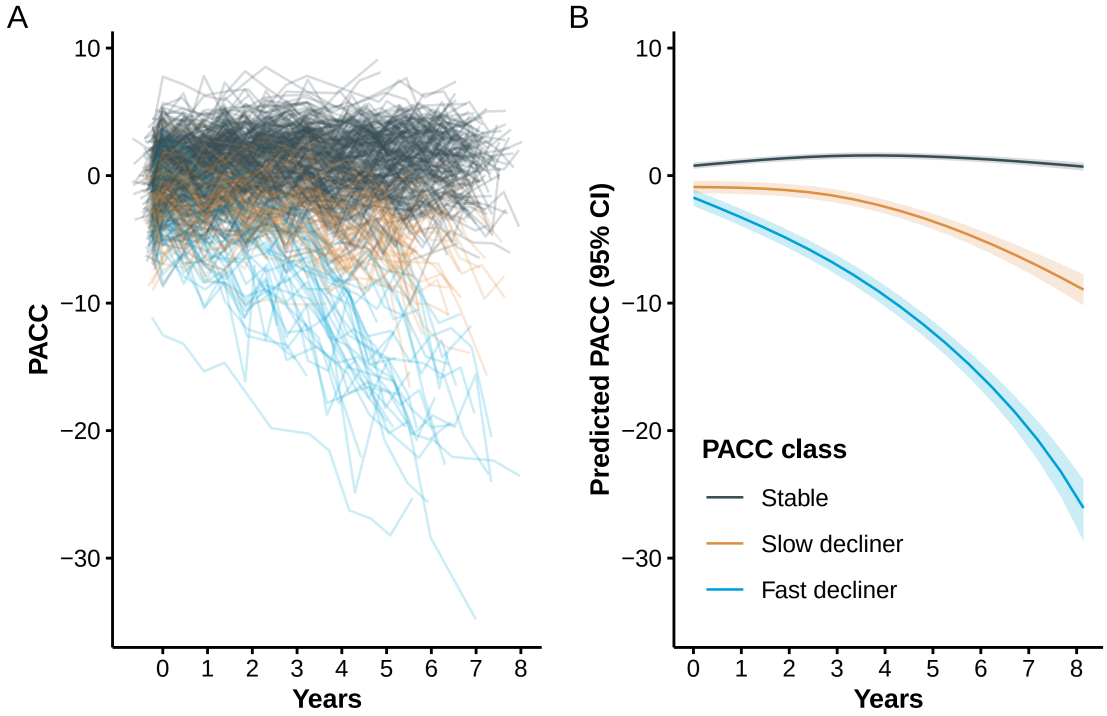

Supplementary Figure S1: **Individual and mean PACC trajectories by latent class model with tau PET.** Left panel (A): Spaghetti plot of individual participant trajectories on the Preclinical Alzheimer Cognitive Composite (PACC), colored by latent class derived from the latent class mixed model (LCMM) which included tau PET as a predictor. Each line represents one participant's observed scores over time. Right panel (B): Estimated mean PACC trajectories for each latent class, with shaded regions indicating 95% confidence intervals. Higher PACC scores indicate better cognitive performance. **Abbreviations:** PET = positron emission tomography.

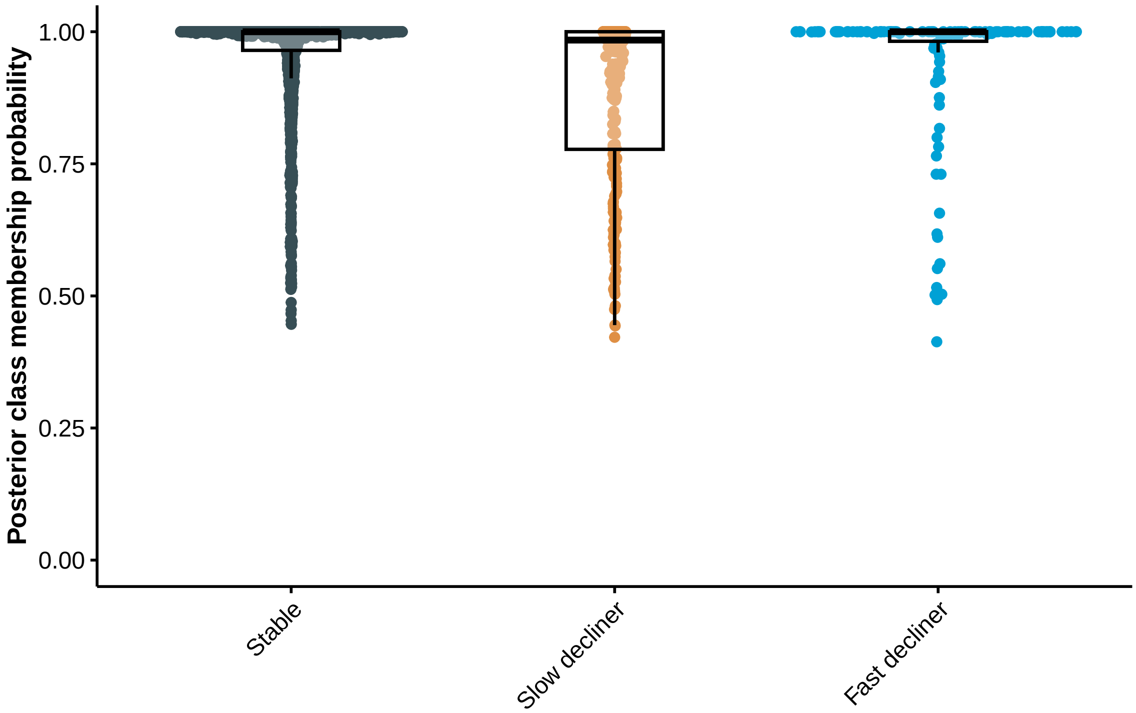

Supplementary Figure S2: **Distribution of posterior class membership probabilities by assigned latent class, indicating model confidence in class assignment.** This plot shows the degree of certainty in class assignments, a good indicator of model reliability. Mean posterior probabilities were 0.95, 0.88, and 0.94 for non-, slow-, and fast decliners. With most participants having high posterior probabilities (> 0.8) for their assigned class, class separation is strong and predictive discrimination is good.

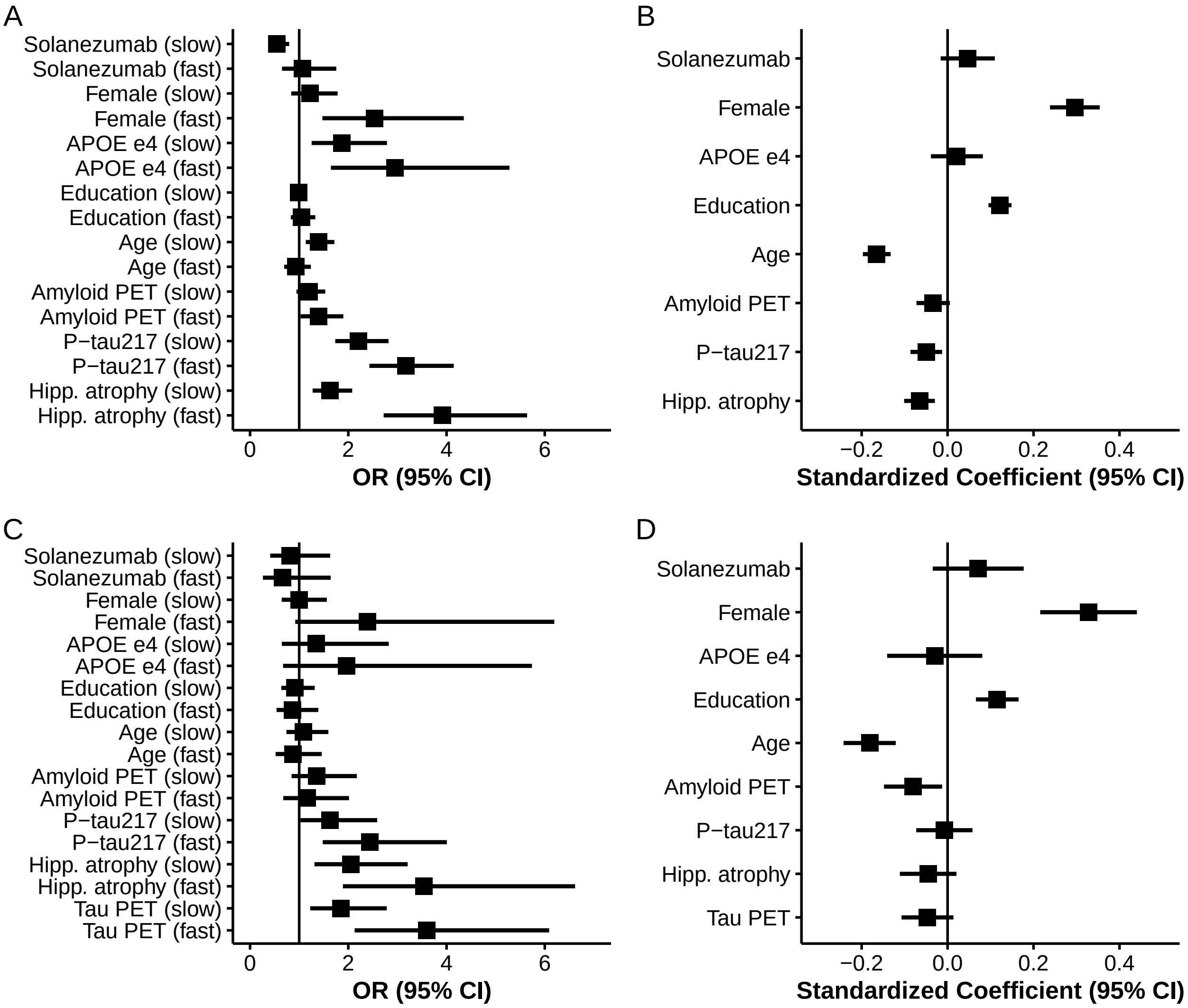

Supplementary Figure S3: **Association of baseline predictors with latent class membership and longitudinal cognitive decline in models with and without tau PET.** Panels A and B display results from the latent class mixed model (LCMM) excluding tau PET; panels C and D show corresponding results from the model including tau PET as an additional predictor. (A, C) Odds ratios (95% CIs) from the LCMM class-membership submodel represent the relative likelihood of belonging to the slow- or fast-declining classes relative to stable class. (B, D) Standardized coefficients (95% CIs) from the LCMM longitudinal submodel show associations between baseline predictors and the Preclinical Alzheimer Cognitive Composite (PACC). All continuous predictors and the PACC outcome were standardized (z-scored) before analysis to enable comparison of effect magnitudes. Higher PACC scores indicate better cognitive performance; thus, larger positive coefficients correspond to slower decline or better preservation of cognition over time.

Model	Parameter	Class membership model			Longitudinal model	
		Class	Estimate (95% CI)	P Value	Estimate (95% CI)	P Value
Base model	Female	(slow)	1.22 (0.84, 1.78)	0.297	0.30 (0.24, 0.35)	<0.001
		(fast)	2.5 (1.5, 4.4)	<0.001		
	Solanezumab	(slow)	0.54 (0.37, 0.80)	0.002	0.047 (-0.016, 0.110)	0.145
		(fast)	1.07 (0.65, 1.75)	0.793		
	APOE e4	(slow)	1.9 (1.3, 2.8)	0.002	0.022 (-0.039, 0.082)	0.482
		(fast)	2.9 (1.6, 5.3)	<0.001		
	P-tau217 (U/ml)	(slow)	0.32 (0.25, 0.41)	<0.001	-0.0071 (-0.0124, -0.0018)	0.008
		(fast)	0.46 (0.35, 0.60)	<0.001		
	Amyloid PET (SUVr)	(slow)	0.26 (0.20, 0.33)	0.135	-0.0072 (-0.0156, 0.0012)	0.091
		(fast)	0.30 (0.22, 0.41)	0.032		
	Age (yrs)	(slow)	6.3 (5.1, 7.8)	0.002	-0.75 (-0.89, -0.60)	<0.001
		(fast)	4.2 (3.1, 5.6)	0.608		
	Education (yrs)	(slow)	2.7 (2.4, 3.0)	0.917	0.33 (0.25, 0.40)	<0.001
		(fast)	2.8 (2.2, 3.6)	0.686		
	Hipp. atrophy (cc^3)	(slow)	1.6 (1.2, 2.0)	<0.001	-0.064 (-0.099, -0.029)	<0.001
		(fast)	3.8 (2.7, 5.5)	<0.001		
Tau PET model	Female	(slow)	1.00 (0.64, 1.56)	0.990	0.33 (0.22, 0.44)	<0.001
		(fast)	2.39 (0.92, 6.19)	0.074		
	Solanezumab	(slow)	0.82 (0.41, 1.63)	0.569	0.071 (-0.035, 0.177)	0.187
		(fast)	0.66 (0.26, 1.64)	0.368		
	APOE e4	(slow)	1.35 (0.65, 2.82)	0.426	-0.030 (-0.141, 0.081)	0.595
		(fast)	1.96 (0.67, 5.74)	0.218		
	P-tau217 (U/ml)	(slow)	0.23 (0.15, 0.37)	0.038	-0.0011 (-0.0105, 0.0084)	0.822
		(fast)	0.35 (0.21, 0.58)	<0.001		
	Amyloid PET (SUVr)	(slow)	0.29 (0.18, 0.47)	0.207	-0.0173 (-0.0318, -0.0027)	0.020
		(fast)	0.25 (0.15, 0.43)	0.582		
	Age (yrs)	(slow)	4.9 (3.4, 7.2)	0.674	-0.82 (-1.10, -0.55)	<0.001
		(fast)	3.9 (2.4, 6.6)	0.599		
	Education (yrs)	(slow)	2.4 (1.7, 3.5)	0.631	0.31 (0.18, 0.44)	<0.001
		(fast)	2.3 (1.4, 3.7)	0.550		
	Hipp. atrophy (cc^3)	(slow)	2.0 (1.3, 3.1)	0.002	-0.044 (-0.109, 0.020)	0.179
		(fast)	3.5 (1.8, 6.5)	<0.001		
	Tau PET (SUVr)	(slow)	0.16 (0.11, 0.24)	0.003	-0.0041 (-0.0094, 0.0012)	0.129
		(fast)	0.31 (0.19, 0.53)	<0.001		

Supplementary Table S2: **Association of baseline predictors (on the *raw* scale) with latent class membership and longitudinal cognitive decline in models with and without tau PET.** The top rows display results from the latent class mixed model (LCMM) excluding tau PET, while the bottom rows show corresponding results from the model including tau PET as an additional predictor. The left side shows odds ratios (95% CIs) from the LCMM class-membership submodel represent the relative likelihood of belonging to the slow- or fast-declining classes relative to stable class. The right side shows coefficients (95% CIs) from the LCMM longitudinal submodel show associations between baseline predictors and the Preclinical Alzheimer Cognitive Composite (PACC). All continuous predictors are on the raw scale. Higher PACC scores indicate better cognitive performance; thus, larger positive coefficients correspond to slower decline or better preservation of cognition over time.

Group	Follow-up (yrs)	Mean PACC un-treated	Standard deviation	Delta (%)	Delta (PACC points)	Power (%)
Decliners	2	-2.80	3.42	20	0.79	93.13
	4	-5.79	5.00	20	1.40	97.66
Stable Amyloid+	2	0.88	2.19	100	0.26	43.88
	4	0.96	2.34	100	0.24	29.54

Supplementary Table S3: **Power for latent class-specific clinical trials.** Effect size estimates were derived from longitudinal models using natural cubic splines (two degrees of freedom) applied to Preclinical Alzheimer Cognitive Composite (PACC) scores among amyloid-positive A4 participants in (a) the stable class and (b) the two decliner classes. The table reports mean PACC and residual variance at 2 and 4 years, representing expected control group trajectories for two potential trial durations. Power was approximated using two-sample t-test calculations assuming 500 participants per group, with 10% attrition at 2 years and 20% at 4 years. Effect size is expressed both as absolute PACC change and as a percentage of the maximum possible improvement, defined as the mean PACC among stable amyloid-negative LEARN participants at the corresponding time point.

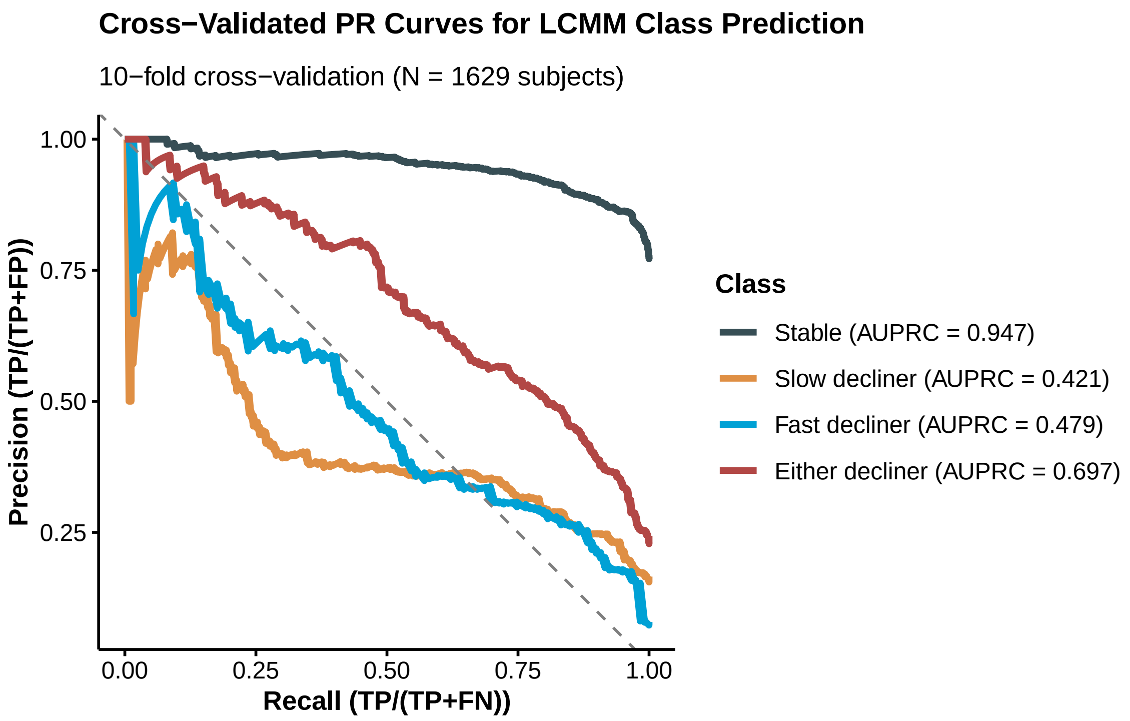

Supplementary Figure S4: **Cross-validated precision-recall curves for latent class membership model without tau PET.** Precision-recall curves summarizing 10-fold cross-validated classification performance of the latent class membership submodel based on baseline demographics and biomarkers, excluding tau PET. Curves depict the ability to distinguish the given class from the other two. The area under the precision-recall curve (AUPRC) quantifies overall predictive performance, emphasizing recall (or sensitivity, TP/(TP+FN)) and precision (TP/(TP+FP)) for less prevalent classes. **Abbreviations:** AUPRC = area under the precision-recall curve; TP = true positive rate; FN = false negative rate; FP = false positive rate.

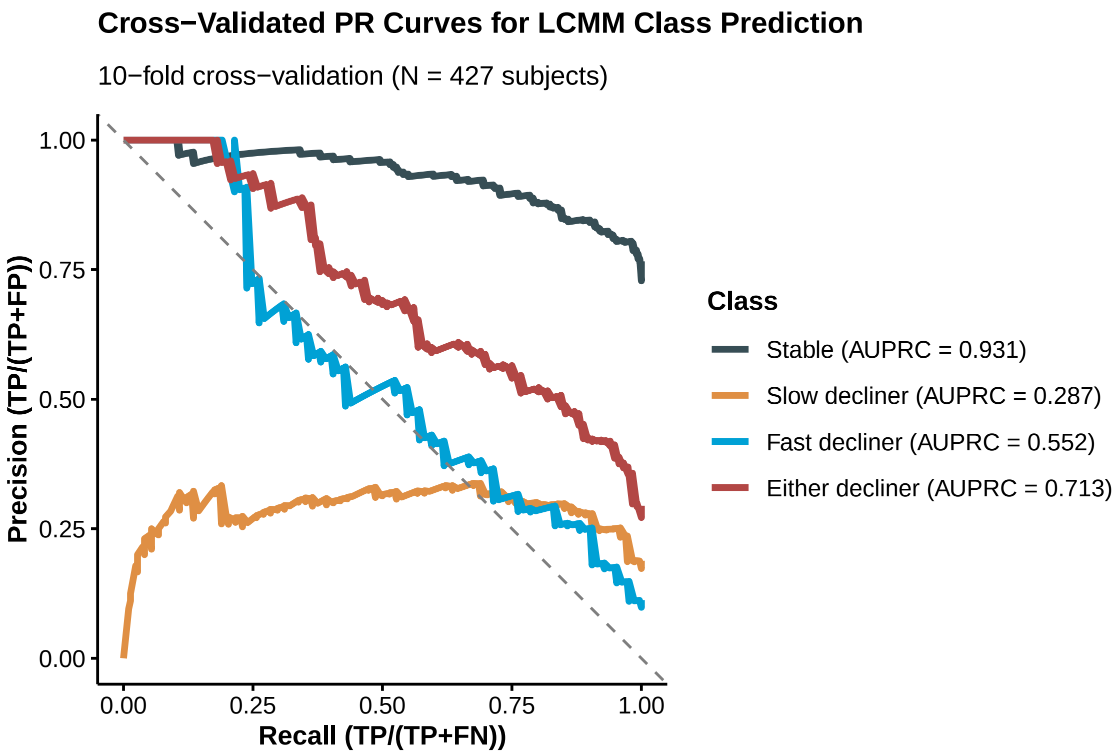

Supplementary Figure S5: **Cross-validated precision-recall curves for latent class membership model with tau PET.** Precision-recall curves summarizing 10-fold cross-validated classification performance of the latent class membership submodel based on baseline demographics and biomarkers, excluding tau PET. Curves depict the ability to distinguish the given class from the other two. The area under the precision-recall curve (AUPRC) quantifies overall predictive performance, emphasizing recall (or sensitivity, TP/(TP+FN)) and precision (TP/(TP+FP)) for less prevalent classes. **Abbreviations:** AUPRC = area under the precision-recall curve; TP = true positive rate; FN = false negative rate; FP = false positive rate.

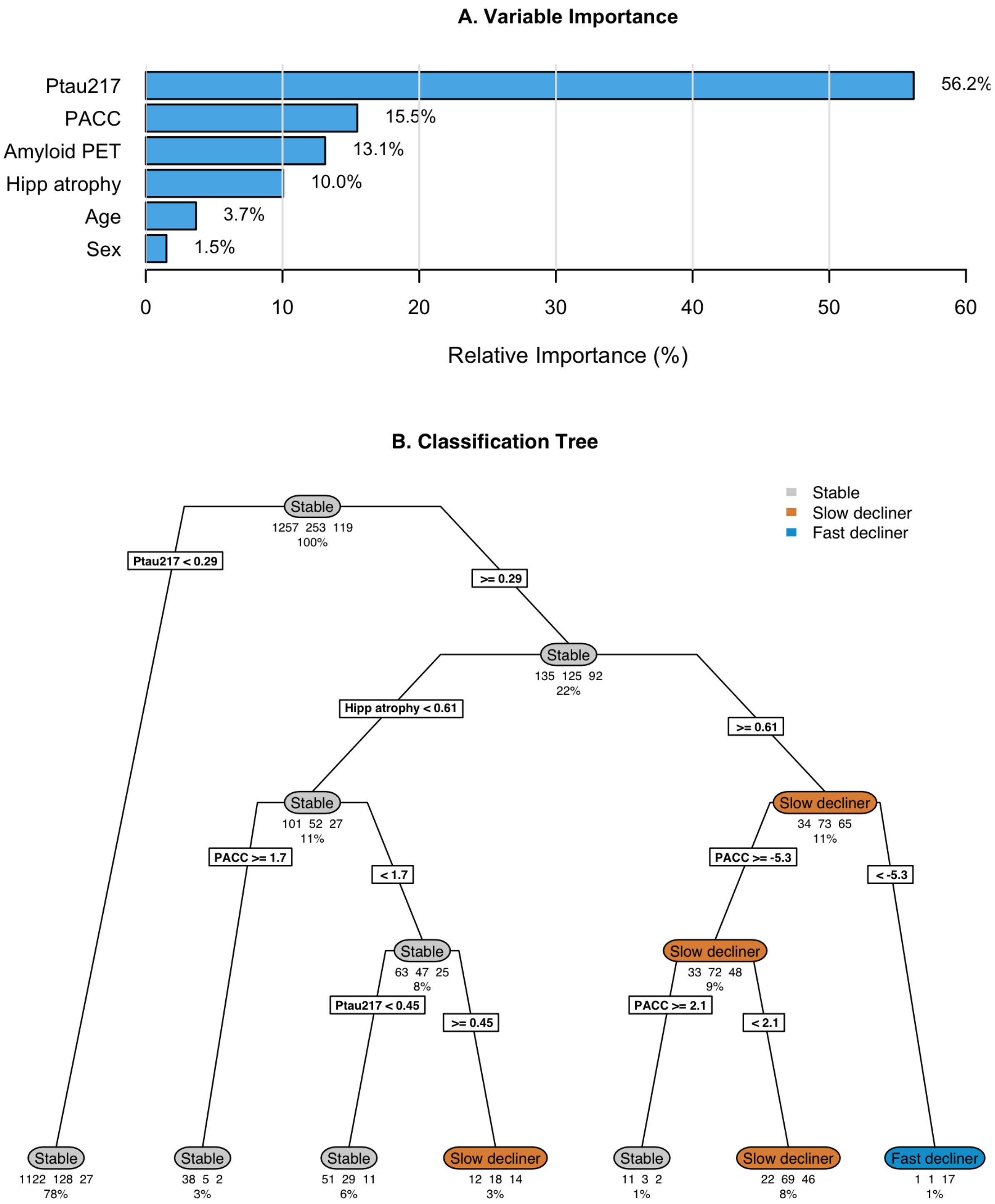

Supplementary Figure S6: **Classification tree analysis for latent class assignment from Base Model.** Panel (A) shows relative importance of baseline predictors for class separation. Panel (B) shows classification tree showing binary decision rules for assigning subjects to latent classes based on predictors. Terminal nodes show predicted class, number of subjects, and percentage of total sample. The optimal tree complexity was determined using 10-fold cross-validation with the 1-standard error rule, resulting in a tree with 6 splits (mean balanced accuracy 0.63, standard error 0.007).

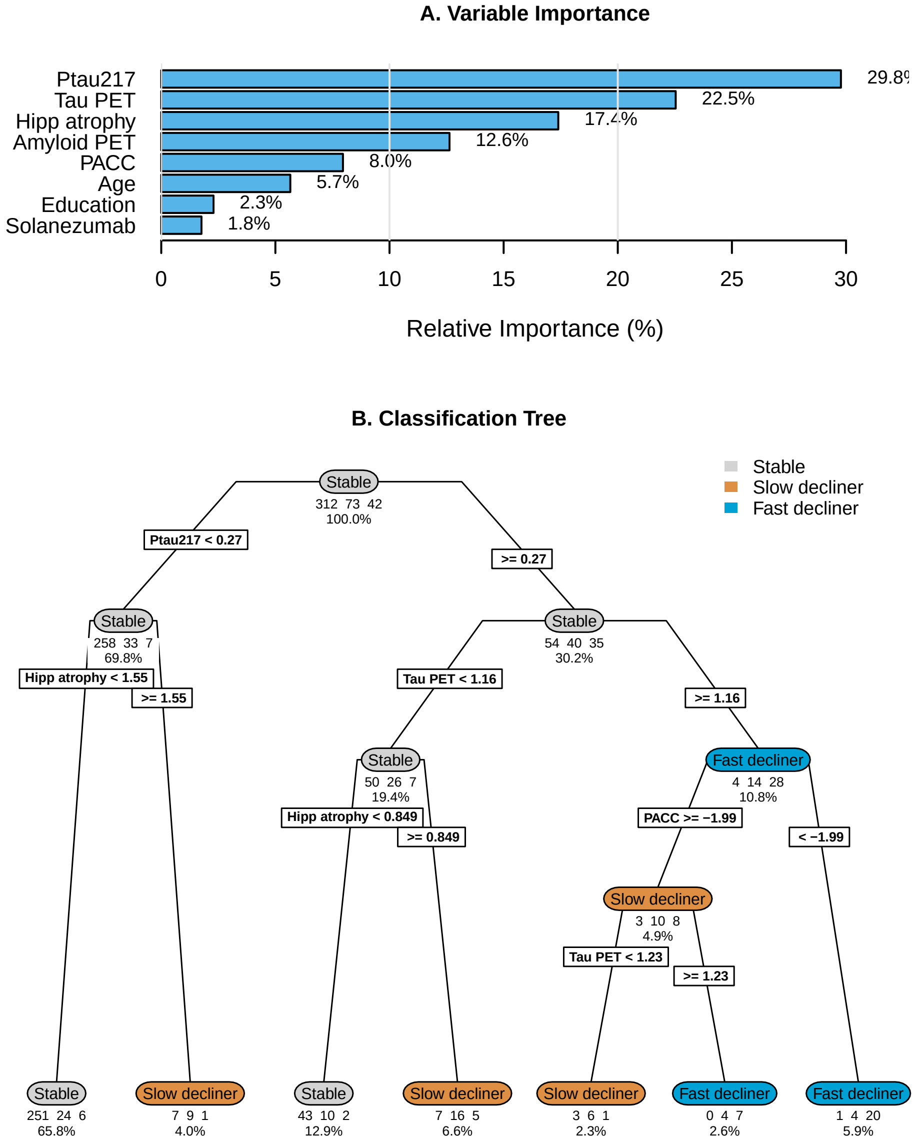

Supplementary Figure S7: **Classification tree analysis for latent class assignment from Tau PET Model.** (A) Relative importance of baseline characteristics for class separation. (B) Classification tree showing binary decision rules for assigning subjects to latent classes based on baseline characteristics. Terminal nodes show predicted class, number of subjects, and percentage of total sample. The optimal tree complexity was determined using 10-fold cross-validation with the 1-standard error rule, resulting in a tree with 6 splits (mean balanced accuracy 0.69 , standard error 0.02).
